# Overweight in childhood of exclusively breastfed infants with a high weight at 5 months

**DOI:** 10.1111/mcn.13057

**Published:** 2020-08-20

**Authors:** Camilla S. Morgen, Melanie W. Larsson, Lars Ängquist, Thorkild I. A. Sørensen, Kim F. Michaelsen

**Affiliations:** ^1^ National Institute of Public Health University of Southern Denmark Copenhagen Denmark; ^2^ Department of Public Health, Section of Epidemiology University of Copenhagen Copenhagen Denmark; ^3^ Department of Nutrition, Exercise and Sports. Faculty of Sciences University of Copenhagen Denmark; ^4^ Department of Nursing and Nutrition, Faculty of Health University College Copenhagen Copenhagen Denmark; ^5^ Novo Nordisk Foundation Center for Basic Metabolic Research, Section of Metabolic Genetics, Faculty of Health and Medical Sciences University of Copenhagen Denmark

**Keywords:** children, cohort study, exclusive breastfeeding, high infant weight, overweight

## Abstract

High infant weight increases the risk of childhood overweight, while breastfeeding may reduce the risk. However, some infants have a very high weight gain even though they are exclusively breastfed. We examined the risk of a high body mass index (BMI) and overweight in childhood for infants ≥2.5 SD above the median weight‐for‐age (WAZ) at age 5 months according to duration of exclusive breastfeeding (≤2, >2 to <4 or ≥4 months). The study is based on 13,401 7‐year‐old and 9,819 11‐year‐old children enrolled into the Danish National Birth Cohort (born 1997–2003). Linear and logistic regression analyses were used to examine the associations while adjusting for presumed confounders including birth weight. The results showed that infants ≥2.5 SD at 5 months, breastfed exclusively ≤2, >2 to <4 or ≥4 months had adjusted odds ratios (ORs) for overweight at age 7 at 3.67 (95% confidence interval [CI] [2.10, 6.43]), 3.42 (95% CI [2.32, 5.04]) and 3.19 (95% CI [1.90, 5.36]) respectively, when compared with infants <2.5 SD WAZ exclusively breastfed ≥4 months. The corresponding results for BMI *z*‐scores were 0.82 (95% CI [0.60, 1.04]), 0.63 (95% CI [0.48, 0.78]) and 0.57 (95% CI [0.38, 0.77]). For the ≥2.5 SD infants, the differences in risk of overweight and BMI according to duration of exclusive breastfeeding were neither significantly different among the 7‐year nor among the 11‐year‐old children. A high infant weight increases the odds of overweight and is associated with a higher BMI in childhood. Whereas the odds and BMI *z*‐scores tended to be lower for those exclusively breastfed longer, the differences were not statistically significant.

**Key messages**

- High infant weight is associated with increased risk of overweight later in life
- Longer duration of exclusive breastfeeding may protect infants from overweight in childhood
- We found that longer duration of exclusively breastfeeding do not protect infants with a very high weight for age from overweight in childhood

## 1 INTRODUCTION

Early infancy and childhood is a sensitive period in relation to development of overweight and obesity later in life (Woo Baidal et al., 2016). The first thousand days of life, that is, from conception to the child reaches 2 years of age, appear to be a critical window for this effect (Aris et al., 2017; Woo Baidal et al., 2016). Systematic reviews show a consistent association between a high weight in the first years of life, and overweight and obesity in childhood and adulthood (Druet et al., 2012; Monteiro et al., 2005; Zheng et al., 2018). The risk of obesity has been shown to be more than three‐fold among infants with rapid weight gain (L. G. Andersen, Holst, Michaelsen, Baker, & Sorensen, 2012; Zheng et al., 2018). The ‘growth acceleration hypothesis’ suggests that the early rapid growth influences the infant's metabolic profile leading to increased susceptibility to later obesity (Singhal et al., 2007). This hypothesis has shown to be particularly relevant for the first 6 months of life (Bjerregaard et al., 2014; Singhal et al., 2007; Young, Johnson, & Krebs, 2012). Compared with formula‐fed infants, breastfed infants gain more fat during the first 6 months (Gale et al., 2012), but breastfed infants have an overall slower growth rate during the first year of life, and they are both shorter and thinner at 12 months (Dewey, Heinig, Nommsen, Peerson, & Lonnerdal, 1993; Patro‐Golab, Zalewski, Polaczek, & Szajewska, 2019). Several studies suggest a protective effect of breastfeeding against overweight and obesity in childhood and adulthood (Arenz, Ruckerl, Koletzko, & von Kries, 2004; Harder, Bergmann, Kallischnigg, & Plagemann, 2005; Horta, Loret de Mola, & Victora, 2015; Weng, Redsell, Swift, Yang, & Glazebrook, 2012; Yan, Liu, Zhu, Huang, & Wang, 2014). However, the randomized cluster controlled trial in Belarus by Kramer and colleagues did not show compelling evidence of a protective effect of breastfeeding on obesity (Kramer et al., 2007). The apparent protective effect of breastfeeding on later risk of obesity in observational studies may be due to lack of adequate control for confounding, especially by the transmitted mother–child predisposition to obesity (Brion et al., 2011; Casazza et al., 2013).

In a prospective population‐based birth cohort study including 3,367 children, van der Willik et al. found that the 406 infants who had a body mass index (BMI)‐for‐age ≥1 SD at 6 months had an increased risk of childhood obesity, defined according to the International Obesity Task Force (IOTF) reference, (Cole, Bellizzi, Flegal, & Dietz, 2000) at 5–6 years of age, but this association was not modified by type of feeding during infancy (van der Willik, Vrijkotte, Altenburg, Gademan, & Kist‐van Holthe, 2015).

A few case reports and a small cohort study have shown that some breastfed infants have an excessive weight gain during exclusive breastfeeding followed by a marked catch‐down when other foods are introduced (Larsson, Lind et al., 2018, Larsson, Larnkjær et al., 2018; Perrella et al., 2016). In the explorative cohort study by Larsson, Lind et al., it is suggested that low milk leptin content could increase appetite and have a role in the excessive weight gain during breastfeeding (Larsson, Lind et al., 2018). However, these studies did not address the long‐term risk of overweight and obesity in these infants.

Clearly, more research is needed to make evidence‐based recommendations for parents and health professionals for children with a high weight gain during exclusive breastfeeding (Larsson, Lind et al., 2018). The Danish National Birth Cohort (DNBC), with its very large sample size and a long follow‐up, provides a unique opportunity to examine whether the risk of overweight or a high BMI in later childhood of infants with a very high infant weight differs by various durations of exclusive breastfeeding. We *hypothesize* that among infants with a high weight in mid‐infancy those who are breastfed exclusively for more than 4 months will have a lower risk of overweight and a lower BMI later in childhood than those breastfed exclusively for a shorter period. We tested this hypothesis in the DNBC by estimating the associations of combined high weight at age 5 months and different durations of exclusive breastfeeding with overweight and BMI in childhood.

## 2 METHODS

### 2.1 A Danish cohort study

The DNBC is a nationwide study of pregnant women and their offspring in which pregnant women from all over the country during the period 1996 through 2002 were invited to participate. They were invited early in the first trimester at their first antenatal visit to the general practitioner. The included women participated in four computer‐assisted telephone interviews, scheduled to take place in gestational Weeks 12 and 30 and when the child was on average 6 and 18 months old. The children were further invited to a 7‐ and 11‐year follow‐up (mean follow‐up time 7.0 and 11.1 years, respectively). The cohort is described in detail elsewhere (Olsen et al., 2001).

### 2.2 Eligibility criteria

**FIGURE 1:**
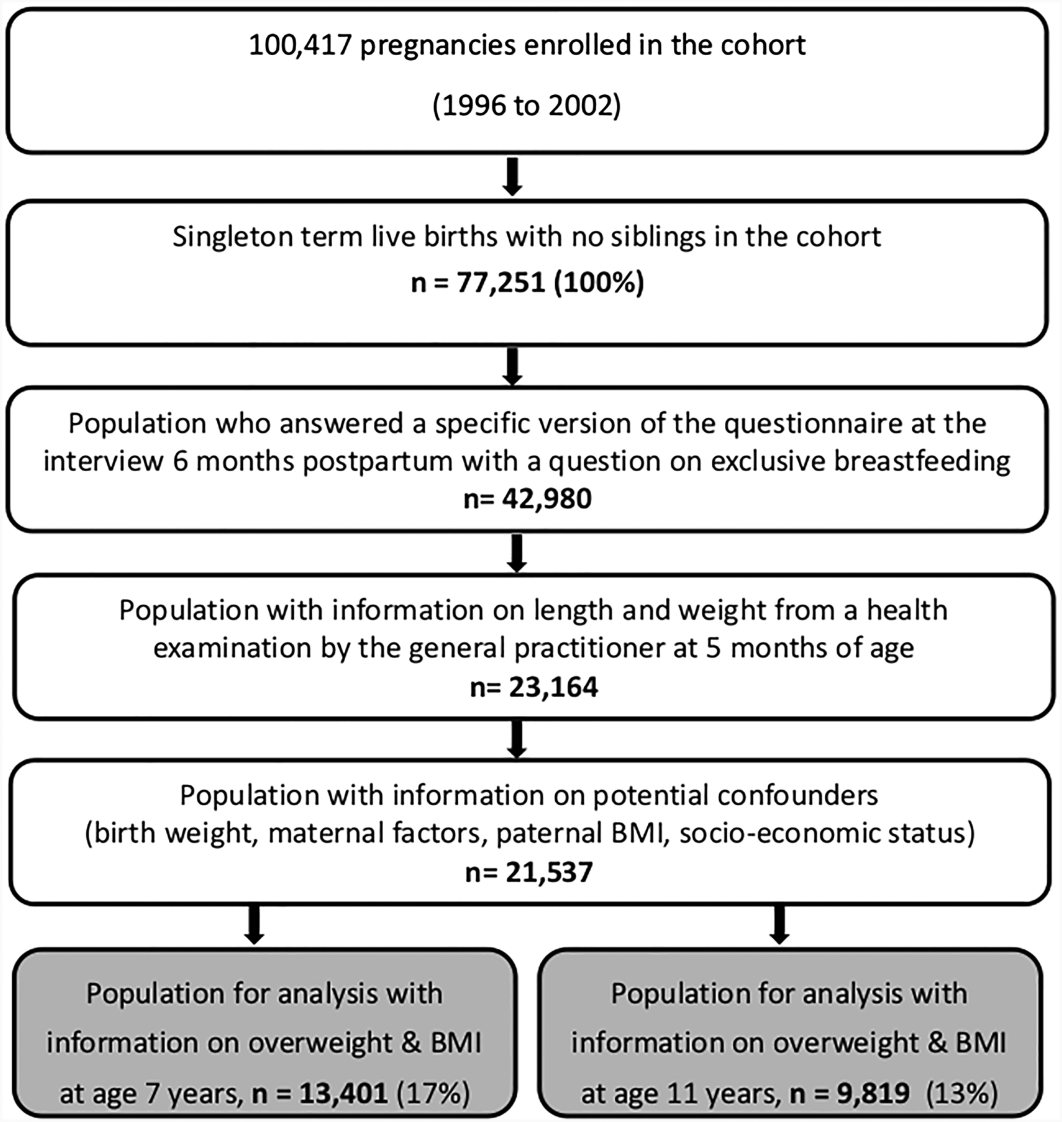
Flow chart defining the samples eligible for analysis based on the Danish National Birth Cohort (DNBC)

A total of 100,417 pregnancies were enrolled in the cohort. However, the necessary criteria for inclusion in the present study reduced the sample size. As a first step, we restricted the sample to the 77,251 live‐born singleton children who were born at term and who had no siblings participating in the cohort (Figure 1). If the mother participated with more than one child, only the first child was included, thereby avoiding mutually dependent observations. Subsequently, we excluded children and parents with missing information on exposure, outcome, and confounders, which was collected in several interviews. Further, owing to an administrative change during the collection of interviews, the question regarding exclusive breastfeeding was slightly changed in a second version of the questionnaire. Therefore, only 72% of the women who participated in the interview 6 months postpartum were asked about duration of exclusively breastfeeding. Eventually, a total of 13,401 participated in the follow‐up, had available information on height, weight, and date of the measurements and were thus were eligible for the analyses with 7‐year follow‐up. A total of 9,819 were eligible for analyses with 11‐year follow‐up (Figure 1). All results regarding 11‐year follow‐up are presented in supplemental tables.

### 2.3 Exposure variables‐exclusive breastfeeding and infant weight‐for‐age

The mothers reported duration of any breastfeeding in interviews 6 and 18 months after birth. The women were asked about their current infant feeding practices including duration of any breastfeeding, exclusive breastfeeding and timing of introduction of complementary foods. In the interview 18 months postpartum, the women were only asked about duration of any breastfeeding. Therefore, we do not have information on exclusive breastfeeding beyond the time for the 6 months interview. Because some of the women had the 6 months interview when the child was 5 months old, we only have unambiguous information on exclusive breastfeeding up until the age of 5 months for the entire group. Children exclusively breastfed longer than 5 months were included in the group with the longest duration of breastfeeding (*n* = 1868 in the 7‐year sample and *n* = 1,409 in the 11‐year sample). We divided exclusive breastfeeding into ≤2, >2 to <4 or ≥4 months and chose ≥4 months as the reference group. Exclusive breastfeeding means no supplement to breast milk beside water and vitamins. The duration of any breastfeeding was used for descriptive purposes and categorized into quartiles (<20, 20 to <32, 32 to <40 and ≥40 weeks). Any breastfeeding is breastfeeding supplemented with formula and/or complementary food.

The infants were weighed, and their lengths measured at age 5 months by the general practitioner or a health nurse. The measurements were recorded in the ‘child's book’, which serves communication between health visitors, the parents and the general practitioner. The parents kept the book, and the mothers reported the measurements of length in the 18 months interview. The 5‐month infant weights were standardized into weight‐for‐age *z*‐scores (WAZ) based on age and sex‐specific cut‐offs from the World Health Organization child growth standards (World Health Organization, 2006).

Based on these standardized weights, we chose a dichotomous indicator for lower versus high infant weight that were then defined based on the threshold score of 2.5 (WAZ < 2.5 vs. ≥ 2.5 WAZ) to capture the infants with a very high WAZ while still having sufficient statistical power to perform the analysis.

### 2.4 Overweight and BMI at ages 7 and 11 years

The mothers reported weight and height at the 7‐year follow‐up in most cases (for 33% measures were taken either by the school doctor, public health nurse or the general practitioner, and for the remaining 67%, measurements were taken by the parents). Information on weight and height at the 11‐year follow‐up were reported by the parents. Overweight and obesity at ages 7 and 11 years were defined according to the IOTF sex‐and age‐specific reference (Cole, Bellizzi, Flegal, & Dietz, 2000). Because less than 1.5% of the children were obese by that definition, children with overweight and obesity were analysed together and referred to as ‘children with overweight’. To remove skewness from the BMI distributions among the children, BMI *z*‐scores were created using an external population reference chart derived through the lambda‐mu‐sigma method (Cole, 1990, Cole & Green, 1992) and using sex‐ and age‐specific (in months) references for standardization (de Onis, Martorell, Garza, & Lartey, 2006).

The reported measurements at age 7 years have been compared with measures of height and weight in a subsample of 1,122 children measured by a school doctor. This validation showed that the percentage of children categorized as being overweight was slightly lower when using the DNBC information than when using the school doctor measurements. However, the validation showed no trends towards increasing differences in weight or height with increasing averages of weight or height between the measurements from school doctors and from the DNBC, suggesting that the disagreements may be treated as random errors (C. Andersen, 2012).

### 2.5 Covariates

Covariates were selected among those available in DNBC if they were considered as putative confounders in the hypothesis under investigation based on their presumed or previously demonstrated association with breastfeeding, high infant weight and with childhood overweight (Blake‐Lamb et al., 2016; Morgen, Angquist, Baker, Andersen & Sorensen, 2018, Morgen, Angquist, Baker, Andersen & Michaelsen 2018). Information on maternal prepregnancy BMI was obtained from the first pregnancy interview in gestational Week 12, and information on paternal BMI was obtained at the fourth interview 18 months postpartum. Information on maternal smoking during pregnancy, maternal socio‐economic status and maternal physical activity during pregnancy was obtained from the first pregnancy interview. Smoking during pregnancy was included as a continuous measure (cigarettes per day). The total number of minutes per week spent on recreational exercise was calculated from the duration of each exercise type and categorized into hours (h) per week (h/week): 0, >0 to <3 and >3. Socio‐economic status was based on the maternal level of education and occupation and was classified as low, medium or high status. The measure was based on the current or most recent job within 6 months, or, if the woman was studying, on the type of education she headed for (Jacobsen, Nohr, & Frydenberg, 2010). Information on gestational weight gain was obtained from the interview 6 months postpartum and was converted into kg per week of the actual gestational length and was included as a continuous measure; because high birth weight is associated with breast feeding, infant weight gain and with later risk of overweight and obesity (Rugholm et al., 2005; Woo Baidal et al., 2016), we also included information on birth weight, which was collected from The Medical Birth Registry (Knudsen et al., 1998).

### 2.6 Statistical analyses

We compared the characteristics of those who were lost to follow‐up with those not lost to follow‐up among those fulfilling the criteria for inclusion in the present study. We also compared the characteristics of the study population according to duration of exclusive breastfeeding. Multiple logistic regression models were used to estimate associations between duration of exclusive breastfeeding and odds ratios (ORs) for overweight, while adjusting for the chosen covariates. Associations between exclusive breastfeeding and BMI in later childhood were examined by linear regression analyses. The associations with breastfeeding and infant weight were conducted as joint effect analyses, where the associations with the two variables were estimated in combination, that is, the corresponding three levels of breastfeeding, each divided by the two levels of infant weight and with the combination of WAZ < 2.5 SD and exclusive breastfeeding ≥4 months as reference. Construction of the variable this way made it possible to compare associations for variable combinations using the common reference category (de Mutsert, Jager, Zoccali, & Dekker, 2009) and thereby identify protective or harmful particular combinations of infant weight and duration of exclusive breastfeeding.

The models were made first without and then with the chosen covariates (maternal prepregnancy BMI, paternal BMI, maternal smoking during pregnancy, maternal physical activity during pregnancy, weekly gestational weight gain, socio‐economic status of the mother and child sex). The covariates were chosen because of their presumed and previously demonstrated association with breastfeeding, high infant weight and childhood overweight (Blake‐Lamb et al., 2016; Morgen et al. 2018; Morgen, et al. 2018). In additional analyses, we included birth weight to allow estimation of the association of weight gain until the infants were 5 months with risk of overweight and BMI in later childhood.

We calculated ORs for overweight at 7 and 11 years of age and regression coefficients for the BMI *z*‐scores (*β* values) at the same ages, both with 95% confidence intervals (CIs). For the linear regression analyses, the linearity of the associations and the approximate consistency with the normal distribution of the residuals from corresponding individual regressions were graphically examined, and no substantial deviations from linearity and no clear violation of the normal distribution assumption were detected.

To examine whether the associations between duration of exclusive breastfeeding and overweight and BMI in childhood were different between infants with a weight below and above 2.5 WAZ, we tested for the corresponding interaction (between the three levels of breastfeeding × the two levels of infant weight). Furthermore, using a test for trend within each of the two infant weight groups, we examined the associations of breastfeeding with the outcomes.

To evaluate the consistency of our results, we repeated all analyses with cut‐off for ‘high weight’ categorized as WAZ ≥ 1 SD. We also conducted analyses with obesity as defined by the IOTF criteria at age 7 and 11 years as the outcome to examine the robustness of including obesity in the category of overweight as the outcome.

## 3 RESULTS

There were small, though statistically significant, differences between those who participated in the first interviews, but were lost to follow‐up, and those who did participate in the 7‐ and the 11‐year follow‐up providing BMI (Table S1). The socio‐economic status was higher, and smoking was less common in the final study population. For the maternal prepregnancy BMI, paternal BMI, birth weight and weight at 5 and 12 months, the absolute differences were very small. The percentages of women who breastfed exclusively >2 to <4 and ≥4 months were slightly larger among those not lost to follow‐up. The included children had a slightly lower median weight at 7 and 11 years, than those not included in the final study population.

In the sample with 7‐year follow‐up (*n* = 13,401), a total of 2.4% of infants had a WAZ ≥ 2.5 SD, and a total of 28.0% were breastfed exclusively for 4 months or more. In the sample with 11‐year follow‐up (*n* = 9,819), the corresponding percentages were 2.2% and 29.1%. Compared with mothers who breastfed their child exclusively for less than 4 months or more, those who did breastfeed that long had a lower BMI, had a partner with a lower BMI, were older, had a higher educational level, higher frequencies of physical activity during pregnancy, lower frequency of smoking and the children had a slightly lower weight at 5 and 12 months (Table 1).

**TABLE 1:** Characteristics of the study population according to exclusive breastfeeding, *n* = 13,401

Duration of exclusive breastfeeding		≤2 months		>2 to <4 months		≥4 months	
	*n*	Mean/median (%)^a^	*n*	Mean/median (%)^a^	*n*	Mean ± SD (%)^a^	*p* value^b^
Maternal pre‐pregnancy BMI (kg/m^2^)	2,408	23.7 (16.0–54.0)	7,236	22.6 (14.5–63.3)	3,757	22.4 (15.2–46.4)	<0.0001
Paternal BMI (kg/m^2^)	2,408	25.0 (16.1–47.9)	7,236	24.7 (16.7–57.9)	3,757	24.7 (17.3–55.6)	<0.0001
Maternal age at birth (years)	2,408	30.4 ± 4.4	7,236	30.6 ± 4.1	3,757	31.8 ± 4.1	<0.0001
Maternal education/occupational class (%)	2,408	100%	7,236	100%	3,757	100%	
Highest level		41.9		57.0		63.8	
Middle level		46.6		37.3		30.2	
Lowest level		11.4		5.8		6.0	<0.0001
Single mother at, 18 months after birth (%)	2,408	2.5	7,236	1.9	3,757	1.4	0.05
Parity (≥1) (%)	2,405	43	7,230	47	3,755	57	<0.0001
Smoking in pregnancy (cig/day,first trimester)	2,408	2.6 ± 4.9	7,236	1.6 ± 3.9	3,757	1.3 ± 3.7	<0.0001
Ever smoking during pregnancy, (yes, %)	2,408	31.5	7,236	20.2	3,757	16.5	<0.0001
Maternal physical exercise during pregnancy (h/week)	2,408	100%	7,236	100%	3,757	100%	
0		71.6		65.6		64.3	
>0 to <3		18.4		23.7		23.2	
≥3		10.1		10.7		12.5	<0.0001
Weekly gestational weight gain (kg)	2,408	0.37 ± 0.15	7,236	0.38 ± 0.13	3,757	0.37 ± 0.13	0.002
Gestational age at birth (days)	2,408	280.1 ± 9.2	7,236	281.5 ± 8.5	3,757	281.0 ± 8.8	0.0004
Birth weight (kg)	2,408	3.6 (1.7–5.7)		3.6 (1.8–5.8)		3.6 (1.8–5.6)^3^	0.02
Gender (boys, %)	2,408	53.3	7,236	52.4	3,757	46.4	<0.0001
Weight at 5 months examination (kg)	2,408	7.9 (4.6–11.1)	7,236	7.7 (3.8–12.4)	3,757	7.7 (4.9–13.0)	<0.0001
High infant weight (WAZ ≥2.5 SD) at 5 months (%)	2,408	2.9	7,236	2.2	3,757	2.3	0.1
Weight at 12 months examination (kg)	2,397	10.5 (6.7–16.0)	7,217	10.2 (6.1–15.7)	3,749	10.0 (6.9–16.8)	<0.0001
Any breastfeeding (weeks, %)	2,182	100%	6,302	100%	3,196	100%	
0 to <20		83.7		17.3		0.2	
20 to <32		7.4		22.9		12.4	
32 to <40		4.3		29.1		26.8	
≥40		4.6		30.7		60.7	<0.0001
Introduction of complementary food (<4 months, %)	2,408	2.5	7,236	1.9	3,757	1.4	<0.0001
Children with overweight^4^ at age 7 (%)	2,408	13.4	7,236	8.7	3,757	8.7	<0.0001

Abbreviations: BMI, body mass index; WAZ, weight‐for‐age.

^a^ Values are percentages for categorical variables, means (SD) for continuous variables with a normal‐like distribution or medians (range) for continuous variables with a skewed distribution.

^b^ *p* value comparing groups according to duration of exclusive breastfeeding, assessed using one‐way ANOVA for continuous variables with a normal‐like distribution, Kruskal–Wallis tests for continuous variables with a skewed distribution and chi‐square tests for categorical variables.

^c^ Birth weight is lowest (3,550 g) in this group but only visible on the second decimal.

^d^ Overweight including categorized according to the International Obesity Task Force reference and obesity is included in the overweight category.

For the infants with WAZ < 2.5 SD, the weight, BMI *z*‐score and percentage of children with overweight at age 7 years were significantly lower with longer duration of exclusive breastfeeding (all *p* < 0.0001) (Table 2). For children with a high WAZ, the values showed the same trend, but were far from statistically significant. Similar patterns were seen for 11‐year weight and BMI, although the percentage of children with overweight did not decrease with longer durations of exclusive breastfeeding for infants with high WAZ (Table S2).

**TABLE 2:** Anthropometric characteristics of the children at age 7 years according to exclusive breastfeeding and weight‐for‐age at 5 months, *n* = 13,401

Duration of exclusive breastfeeding			≤2 months	>2 to <4 months	≥4 months^a^		
	*n* ^a^	Weight 5 months^b^	Mean/median (%)^c^	Mean/median (%)^c^	Mean/median (%)^c^	*p* value^d^	
	13,085/316					
Height at 7 years (cm)		≥2.5 SD	130.9 (116.9–142.0)	130.3 (116.0–142.0)	130.5 (116.5–141.0)	0.3
		<2.5 SD	125.8 (110.0–148.0)	125.5 (110.0–150.0)	125.3 (110.0–142.0)	0.02
Weight at 7 years (kg)		≥2.5 SD	30.5 (19.8–52.0)	29.0 (18.9–42.7)	28.5 (19.8–45.0)	0.2
		<2.5 SD	25.1 (14.0–54.0)	24.6 (14.0–48.0)	24.5 (15.5–57.0)	<0.0001
BMI *z*‐score at 7 years^e^		≥2.5 SD	1.01 ± 1.1	0.76 ± 0.9	0.72 ± 1.0	0.2
		<2.5 SD	0.07 ± 1.1	−0.06 ± 1.0	−0.07 ± 0.9	<0.0001
Children with overweight at 7 years (%)^f^		≥2.5 SD	32.9	28.5	27.3	0.7
		<2.5 SD	12.8	8.3	8.2	<0.0001

Abbreviation: BMI, body mass index.

^a^ The numbers are infants <2.5 SD weight‐for‐age at 5 months/infants ≥2.5 SD weight‐for‐age at 5 months.

^b^ High infant weight (at 5 months) is defined as ≥2.5 SD above the median weight‐for‐age (WHO).

^c^ Values are percentages for categorical variables, means (SD) for continuous variables with a normal‐like distribution, or medians (range) for continuous variables with a skewed distribution.

^d^ *p* value comparing groups according to duration of exclusive breastfeeding, assessed using one‐way‐ANOVA for continuous variables with a normal‐like distribution, Kruskal–Wallis test for continuous variables with a skewed distribution and chi‐square tests for categorical variables.

^e^ BMI *z*‐scores are calculated according to the LMS method.

^f^ Overweight including obesity is categorized according to the International Obesity Task Force reference and obesity is included in the overweight category.

In the logistic regression analyses, we found that infants with a high WAZ at 5 months who were breastfed exclusively for ≤2, >2 to <4 or ≥4 months had adjusted ORs for overweight at age 7 at 3.67 (95% CI [2.10, 6.43]), 3.42 (95% CI [2.32, 5.04]) and 3.19 (95% CI [1.90, 5.36]), respectively, when compared with infants with a WAZ < 2.5 SD infants breastfed ≥4 months (Table 3). Although the adjusted ORs decreased stepwise with longer durations of breastfeeding, the interaction between duration of exclusive breastfeeding and high infant WAZ was not significant (*p* = 0.87). Similar results were found for age 11 with stepwise decrease in ORs with longer durations of breastfeeding, but with overlapping CIs and a nonsignificant interaction (Table S3).

**TABLE 3:** Associations between duration of exclusive breastfeeding, infant weight‐for‐age and overweight at age 7 years, *n* = 13,401

		Overweight^a^ at age 7 years					
Exposure	Breastfeeding (month)	OR crude	95% CI	*p* value^b^	OR adjusted^c^	95% CI	*p* value^b^
Weight‐for‐age at 5 months <2.5 SD	≤2	1.65	(1.39, 1.95)	—	1.22	(1.01, 1.46)	—
	>2 to <4	1.01	(0.87, 1.16)	—	0.96	(0.82, 1.11)	—
	≥4	1.00	—	—	1.00	—	—
Weight‐for‐age at 5 months ≥2.5 SD	≤2	5.48	(3.28, 9.14)	—	3.67	(2.10, 6.43)	—
	>2 to <4	4.46	(3.09, 6,42)	—	3.42	(2.32, 5.04)	—
	≥4	4.20	(2.59, 6.81)	0.67	3.19	(1.90, 5.36)	0.87

Abbreviations: BMI, body mass index; CI, confidence interval; OR, odds ratio.

^a^ Overweight is categorized according to the International Obesity Task Force reference.

^b^ *p* values are for the interaction between exclusive breastfeeding and infant weight‐for‐age.

^c^ Adjusted for maternal prepregnancy BMI, paternal BMI, maternal smoking during pregnancy (continuous), maternal physical activity during pregnancy (three levels), weekly gestational weight gain, socio‐economic status of the mother (three levels), child sex and birth weight (continuous).

In the linear regression analyses, we found increased BMI *z*‐scores at age 7 years; 0.82 (95% CI [0.60, 1.04]), 0.63 (95% CI [0.48, 0.78]) and 0.57 (95% CI [0.38, 0.77]) for infants with a high WAZ breastfed exclusively for ≤2, >2 to <4 or ≥4 months, when compared with infants with a WAZ < 2.5 SD, who were breastfed exclusively for ≥4 months (Table 4). Whereas the BMI *z*‐scores decreased stepwise with longer durations of exclusive breastfeeding, the adjustment for potential confounders including birth weight reduced the estimated differences (Table 4). The interaction between duration of exclusive breastfeeding and BMI *z*‐score at age 7 was not significant (*p* = 0.35). Comparable results were found for BMI *z*‐scores at age 11 (Table S4).

**TABLE 4:** Associations between duration of exclusive breastfeeding, infant weight‐for‐age and BMI *z*‐score at age 7 years, *n* = 13,401

		BMI *z*‐score^a^ at age 7 years					
Exposure	Breastfeeding (month)	*β* crude	95% CI	*p* value^b^	*β* adjusted^c^	95% CI	*p* value^b^
Weight‐for‐age at 5 months <2.5 SD	≤2	0.14	(0.09, 0.19)	—	0.03	(−0.02, 0.08)	—
	>2 to <4	0.02	(−0.03, 0.05)	—	−0.01	(−0.05, 0.02)	—
	≥4	0.00	—	—	0.00	—	—
Weight‐for‐age at 5 months ≥2.5 SD	≤2	1.08	(0.85, 1.32)	—	0.82	(0.60, 1.04)	—
	>2 to <4	0.84	(0.68, 0.99)	—	0.63	(0.48, 0.78)	—
	≥4	0.79	(0.59, 1.00)	0.21	0.57	(0.38, 0.77)	0.35

Abbreviations: BMI, body mass index; CI, confidence interval.

^a^ BMI *z*‐score was calculated according to the LMS method.

^b^ *p* values are for the interaction between exclusive breastfeeding and infant weight‐for‐age.

^c^ Adjusted for maternal prepregnancy BMI, paternal BMI, maternal smoking during pregnancy (continuous), maternal physical activity during pregnancy (three levels), weekly gestational weight gain, socio‐economic status of the mother (three levels) and child sex and birth weight (continuous).

Analyses based on only the high WAZ infants also resulted in a stepwise decrease, although statistically insignificant, in odds of overweight and a lower BMI *z*‐score at both ages 7 and 11 years for the infants breastfed exclusively for longer durations (Tables S5–S6). Similar nonsignificant trend results were found also for the lower WAZ infants (data not shown).

To test the consistency of our results, we conducted analyses with a lower cut‐off for ‘high weight‐for‐age’ at WAZ 1 SD, which revealed similar results as for WAZ 2.5 SD (data not shown). Analyses of obesity as defined by IOTF at age 7 years as the outcome showed similar pattern of results as for overweight. The ORs were somewhat higher, but with much wider CIs, and the interaction between a high WAZ and duration of exclusive breastfeeding was not significant (*p* = 0.44, data not shown). The samples of obese 11‐year olds were too small to conduct analyses with interaction terms (data not shown).

## 4 DISCUSSION

In this large prospective cohort study, we confirmed that infants with a high weight at 5 months have increased odds of having overweight and a higher BMI later in childhood, but duration of exclusive breastfeeding until that age had limited influence of these associations. Whereas the odds for overweight and BMI *z*‐scores tended to be lower for those exclusively breastfed for longer durations, these association were not statistically significant. The interaction analyses suggested that the possible decrease in odds for having overweight with longer durations of exclusive breastfeeding was not truly different for the infants having a normal to high WAZ below 2.5 SD versus those with a high WAZ at 5 months.

Our study has some strengths and limitations that needs to be considered to qualify the conclusions and the comparisons with other studies. An important strength is the prospective design and the large number of mother–child pairs with available information on exclusive breastfeeding, and available values on height and weight at birth, 5 months, 7 and 11 years. Second, we had la arge panel of covariates with putative confounding effects, including important risk factors for childhood overweight such as maternal BMI, paternal BMI, birth weight, socio‐economic status and smoking during pregnancy. The adjustment for birth weight in the statistical analyses of the infant weight at 5 months is important because the it implies that the results reflect the weight gain during the first 5 months. The long follow‐up period allows us to assess possible long‐term effects and to compare the results for outcomes at ages 7 and 11 years.

On the other hand, there are also limitations to be considered. The loss to follow‐up may cause unpredictable bias in the estimated associations. Generally, women from higher socio‐economic groups are overrepresented in the DNBC (Jacobsen, Nohr, & Frydenberg, 2010), and the women who participated in follow‐up did more often breastfeed exclusively for longer durations. Compared with all the participants in the 7‐ and 11‐year follow‐up, children in the final study samples were a bit lighter at age 11, but only minimally lighter at age 7 years. The attrition of the study population may imply various risk of selection biases of the final results, but the distributions of the key variables of the final study samples did not raise suspicion of such biases. Also, we cannot rule out that the selection into the cohort in general may have affected our results. We find it too speculative to try to predict the direction and magnitudes of the various possible biases, which of course implies needs for some caution in drawing conclusions.

A further limitation is the lack of validation of the measurements of infant weight and length, which were taken by the health professionals. We consider this to be minor, and we believe that any possible errors in the measurements will cause nondifferential bias. Another possible limitation is that parental reporting of height and weight that may lead to overreporting of low height and underreporting of high weight (Elgar, Roberts, Tudor‐Smith, & Moore, 2005; Goodman, Hinden, & Khandelwal, 2000). Because the validation study of the 7 year values did not show systematic errors in the higher values (C. Andersen, 2012), we do expect the estimates of BMI in childhood to be sufficiently accurate to support our conclusions.

In a prospective study by van der Willik and colleagues, the authors found that exclusively breastfed overweight infants are at the same risk of childhood overweight as formula‐fed overweight infants (van der Willik, Vrijkotte, Altenburg, Gademan, & Kist‐van Holthe, 2015). We did not examine exactly the same research question, but we did not find a difference in the associations between duration of exclusive breastfeeding and later overweight and BMI for the infants with either a high or a lower weight. The differences between our study and the study by van der Willik et al. is that we have based our conclusions on a higher cut‐off, a larger sample size and a longer follow‐up. Analyses of data from the DNBC have previously shown that children who were breastfed (any breastfeeding) for longer durations had slower growth the first year of life, but duration of any breastfeeding was not associated with overweight or BMI at age 7 or 11 years, when adjusted for relevant covariates (Morgen et al., 2018). The present study does not preclude that there may be some beneficial effects of longer durations of *exclusive* breastfeeding, but our results suggest that all infants with a high weight in infancy are at increased odds of having overweight later in life.

However, efforts are still needed to understand why some infants have a high weight‐for‐age during exclusive breastfeeding. Studies on exclusively breastfed infants with a high weight or a rapid weight gain are sparse, and there is limited information about potential causes for the rapid weight gain. A study from Argentina examined breast milk macronutrient content among 65 infants younger than 6 months with a WAZ > 2.5 SD, who were exclusively breastfed (Saure, Armeno, Barcala, Giudici, & Mazza, 2017). This analysis showed that the fat content in the breast milk among mothers of the heavy infants was lower, while the protein content was not different from that in the general population. This is in line with findings from a study comparing breast milk composition and growth, in which the authors found that a high fat percentage was associated with lower weight gain and lower BMI (Prentice et al., 2016). Hormones related to growth, satiety and energy metabolism found in breast milk, such as insulin, leptin and adiponectin, have in some studies been associated with growth in infancy (Eriksen, Christensen, Lind, & Michaelsen, 2018; Fields, Schneider, & Pavela, 2016; Lind, Larnkjaer, Molgaard, & Michaelsen, 2018).

The findings from the present study suggest that also infants with a high WAZ, conditional on birth weight, have increased odds of overweight, or higher BMI, later in childhood even though they were breastfed exclusively for a relatively long period. In a small cohort studied by Larsson, Lind et al (2018), infants with excessive weight gain during exclusive breastfeeding had a subsequent marked catch‐down when other foods were introduced. The average WAZ at 5–6 months was considerably higher than in our study, about 3 SD. In that cohort study, breast milk leptin was low, suggesting an effect on appetite regulation. Thus, the mechanisms responsible for the high weight during exclusive breastfeeding in that cohort might be different from what is seen in the present large cohort. However, we know of no study that has tried to intervene during exclusive breastfeeding to reduce a high early weight gain, and there is no other evidence to support such intervention in view of the many positive effects of exclusive breastfeeding (Victora et al., 2016). It may be important to consider how and when complementary food is introduced to these exclusively breastfed infants with high weight gain. It is important to strive for the optimal composition of the early diet and responsive feeding and to initiate relevant physical stimulation, in accordance with current recommendations (Fewtrell et al., 2017).

We conclude that infants with a high weight gain during the first 5 months have much higher odds for developing overweight and have an overall higher BMI at age 7 and 11 years. Although these associations tended to be slightly weaker by a prolonged period of exclusive breastfeeding, the differences were not significantly different. A longer duration of exclusively breastfeeding does not seem to protect infants with a high weight from overweight in childhood.

## CONFLICTS OF INTEREST

The authors declare that they have no conflicts of interest.

## CONTRIBUTIONS

KFM conceived the study. CSM, KFM, LÄ and TIAS developed the study design. TIAS and KFM were involved in the data collection. CSM and LÄ carried out the statistical analyses. CSM, MWL and KFM conducted the literature search. CSM drafted the manuscript. All authors were involved with the interpretation of results, critical revision of the manuscript and approved the final submitted version.

## Supporting information


**Table S1.** Characteristics of the full study population and of the selected sample with information on exposure and outcome variables

**Table S2.** Anthropometric characteristics of the children at 11 years according to tertiles of exclusive breastfeeding and weight for age at 5 months, *n* = 9,819

**Table S3.** Associations between duration of exclusive breastfeeding, infant weight and overweight at age 11 years, *n* = 9,819

**Table S4.** Associations between duration of exclusive breastfeeding, infant weight and BMI z‐score at age 11 years

**Table S5.** Associations between duration of exclusive breastfeeding, overweight1 and BMI z‐score at age 7 years for infants ≥2.5 SD weight for age at 5 months, *n* = 316.

**Table S6.** Associations between duration of exclusive breastfeeding, overweight1 and BMI z‐score at age 11 years for infants ≥2.5 SD weight for age at 5 months, *n* = 214.
