# Supplementary material for: Overweight in childhood of exclusively breastfed infants with a high weight at 5 months
Source: Matern Child Nutr. 2020 Aug 20;17(1):e13057. doi: 10.1111/mcn.13057 (PMC7729543; doi:10.1111/mcn.13057)
Supplement: Supplementary file 1 — Table S1. Characteristics of the full study population and of the selected sample with information on exposure and outcome variables [file MCN-17-e13057-s001.pdf]

Supplementary Table 1. Characteristics of the full study population and of the selected sample with information on exposure and outcome variables

Characteristics	Full population		7 year BMI sample			11 year BMI sample		
	n	Median/ Mean/ %[1]	n	Median/ Mean/ %[1]	*p-value*[2]	n	Median/ Mean/ %[1]	*p-value*[3]
**Maternal pre-pregnancy BMI,** (kg/m^2)	75,941	22.6 (14.2-64.4)	13,401	22.7 (14.5-63.3)	0.006	9,819	22.6 (15.5-63.3)	0.06
**Paternal BMI,** (kg/m^2)	53,622	24.8 (14.9-57.9)	13,401	24.7 (16.1-57.9)	0.3	9,819	24.7 (16.7-57.9)	0.01
**Maternal education/occupational class,** (%)	76,947	100%	13,401	100%		9,819	100%	
Highest level		51.9		56.2			59.4	
Middle level		38.1		37.0			34.5	
Lowest level		10.1		6.9	<0.001		6.1	<0.001
**Single mother at, 18 months after birth**, (%)	56,277	4.1	13,400	1.9	<0.001	9,819	1.4	<0.001
**Smoking in pregnancy,** (cig/day,1st trimester)	77,028	2.2 ± 4.7	13,401	1.7 ± 4.1	<0.001	9,819	1.5 ± 3.8	<0.001
**Ever smoking during pregnancy,** (yes %)	77,207	27.6	13 401	21.1	<0.001	9,819	19.3	<0.001
**Maternal physical exercise during pregnancy** (h/week)	72,568	100%		100%		9,819	100%	
0		69.9	13,401	66.3			65.3	
0>-3		19.5		22.6			23.1	
≥3		10.6		11.1	<0.001		11.5	<0.001
**Weekly gestational weight gain,** (kg)	59,526	0.38 ± 0.14	13,401	0.37 ± 0.14	<0.001	9,819	0.37 ± 0.13	<0.001
**Gestational age at birth,** (days)	77,251	281.3 ± 8.9		281.5 ± 8.7	0.4	9,819	281.3 ± 8.7	0.35
**Birth weight,** (kg)	76,900	3.6 (1.5-6.0)	13,401	3.6 (1.7-5.8)	0.002	9,819	3.6 (1.8-5.8)[4]	0.004
**Gender** Boys, (%)	77,251	51.1	13,401	50.9	0.6	9,819	49.6	0.002
**Weight at 5 months examination,** (g)	48,422	7.8 (3.1-13.2)	13,401	7.8 (3.8-13.0)	0.3	9,819	7.7 (3.8-13.0)	0.004
**Weight at 12 months examination,** (g)	45,421	10.2 (4.1-18.9)	13,363	10.2 (6.1-16.8)	0.008	9,794	10.2 (6.1-16.0)	0.002
**Exclusive breastfeeding,** (%)	42,980	100%	13,401	100%		9,819	100%	
≤2 months		21.9		18.0			16.5	
>2- <4 months		51.8		54.0			54.4	
≥4 months		26.9		28.0	<0.001		29.1	< 0.001
**Introduction of complementary food** <4 months (%)	58,024	8.5		7.0	<0.001	9,431	6.7	<0.001
**Weight at age 7 years**[5]	42,180	24.5 (12.0-68.0)	13,401	24.3 (14.0-57.0)	<0.001	9,398	24.1 (12.5-54.0)	<0.001
**Weight at age 11 years**[5]	28,430	39.0 (20.0-98.0)	9,532	37.0 (21.0-97.0)	<0.001	9,819	37.0 (21.0-97.0)	<0.001

[1]Values are percentages for categorical variables, means (SD) for continuous variables with a normal distribution, or medians (range) for continuous variables with a skewed distribution, [2]P-value comparing the population available for analyses with 7 year BMI compared to the larger group, who had some information, but were not included in the final analyses, assessed using one-way-ANOVA for continuous variables with a normal distribution, Kruskal-Wallis test for continuous variables with a skewed distribution and chi-square tests for categorical variables, [3]P-value comparing the population available for analyses with 11 year BMI compared to the group who had some information, but were not included in the final analyses, [4] Birth weight is lowest (3550g) in this group but only visible on the 2nd decimal, [5]The number with available weight information at age 7 and 11 years is higher than the n in the 7 and 11 year BMI sample, since these analytical samples were based on a study population with information on height, date of the measurements and all relevant covariates.
