# Supplementary material for: Overweight in childhood of exclusively breastfed infants with a high weight at 5 months
Source: Matern Child Nutr. 2020 Aug 20;17(1):e13057. doi: 10.1111/mcn.13057 (PMC7729543; doi:10.1111/mcn.13057)
Supplement: Supplementary file 2 — Table S2. Anthropometric characteristics of the children at 11 years according to tertiles of exclusive breastfeeding and weight for age at 5 months, n = 9,819 [file MCN-17-e13057-s002.pdf]

Supplementary Table 2. Anthropometric characteristics of the children at 11 years according to tertiles of exclusive breastfeeding and weight for age at 5 months, n= 9,819

Duration of exclusive breastfeeding	n[1]	Weight 5 months[2]	≤2 months<br>Mean ± SD/%[3]	>2- <4 months<br>Mean ± SD/%[3]	≥4 months<br>Mean ± SD/%[3]	*p-value*[4]
**Height at 11 years,** (cm)	9,605 / 214	≥2.5 SD	157.7 (140.0-170.0)	153.8 (138.0-169.0)	152.5 (140.0-164.0)	0.002
		<2.5 SD	149.5 (102.0-148.0)	149.1 (100.0-150.0)	149.1 (102.0-142.0)	0.1
**Weight at 11 years,** (kg)		≥2.5 SD	47.7 (32.0-72.0)	44.7 (30.0-64.0)	43.0 (30.0-60.0)	0.04
		<2.5 SD	39.5 (15.0-54.0)	38.3 (14.1-43.0)	38.2 (12.5-42.4)	<0.0001
**BMI z-score[5] at 11 years**		≥2.5 SD	0.68 ± 1.0	0.64 ± 0.8	0.51 ± 0.8	0.5
		<2.5 SD	0.10 ± 1.1	-0.05 ± 0.9	-0.07 ± 1.0	<0.0001
**Overweight[6] at age 11**, (%)		≥2.5 SD	19.5	20.0	20.7	0.99
		<2.5 SD	13.0	7.8	8.6	<0.0001

[1]The numbers are infants < 2.5 SD weight for age at 5 months /infants ≥ 2.5 SD weight for age at 5 months, [2]High infant weight (at 5 months) is defined as ≥ 2.5 SD above the median weight-for-age (WHO), [3] Values are percentages for categorical variables, means (SD) for continuous variables with a normal-like distribution, or medians (range) for continuous variables with a skewed distribution, [4] P-value comparing groups according to duration of exclusive breastfeeding, assessed using one-way-ANOVA for continuous variables with a normal-like distribution, Kruskal Wallis test for continuous variables with a skewed distribution and chi-square tests for categorical variables, [5]BMI z-score are calculated according to the LMS method, [6]Overweight is categorized according to the International Obesity Taskforce reference and obesity is included in the overweight category.
