# Supplementary material for: Overweight in childhood of exclusively breastfed infants with a high weight at 5 months
Source: Matern Child Nutr. 2020 Aug 20;17(1):e13057. doi: 10.1111/mcn.13057 (PMC7729543; doi:10.1111/mcn.13057)
Supplement: Supplementary file 3 — Table S3. Associations between duration of exclusive breastfeeding, infant weight and overweight at age 11 years, n = 9,819 [file MCN-17-e13057-s003.pdf]

Supplementary Table 3. Associations between duration of exclusive breastfeeding, infant weight and overweight at age 11 years, n = 9,819

	Overweight[1] at age 11 years						
**Exposure**	**Breastfeeding**	**OR**	**95% CI**	*p-value*[2]	**OR adjusted**[3]	**95% CI**	*p-value*[2]
Weight for age at 5 months <2.5 SD	≤2 months	1.59	(1.30, 1.93)		1.26	(1.00, 1.58)	
	>2- <4 months	0.89	(0.75, 1.05)		0.97	(0.81, 1.17)	
	≥ 4 months	1.00	-		1.00	-	
Weight for age at 5 months ≥2.5 SD	≤2 months	2.57	(1.17, 5.63)		5.50	(2.72, 11.13)	
	>2-<4 months	2.65	(1.65, 4.26)		3.34	(2.08, 5.37)	
	≥4 months	2.77	(1.45, 5.29)	0.39	3.16	(1.66, 6.04)	0.63

[1]Overweight is categorized according to the International Obesity Task Force reference,[2] P-values are for the interaction between exclusive breastfeeding and infant weight , [3]Adjusted for: maternal pre-pregnancy BMI, paternal BMI, maternal smoking during pregnancy (continuous), maternal physical activity during pregnancy (3 levels), weekly gestational weight gain, socio-economic status of the mother (3 levels) and child sex and birth weight (continuous).
