# Supplementary material for: Overweight in childhood of exclusively breastfed infants with a high weight at 5 months
Source: Matern Child Nutr. 2020 Aug 20;17(1):e13057. doi: 10.1111/mcn.13057 (PMC7729543; doi:10.1111/mcn.13057)
Supplement: Supplementary file 4 — Table S4. Associations between duration of exclusive breastfeeding, infant weight and BMI z‐score at age 11 years [file MCN-17-e13057-s004.pdf]

Supplementary Table 4. Associations between duration of exclusive breastfeeding, infant weight and BMI z-score at age 11 years

	BMI z-score[1] at age 11 years						
**Exposure**	**Breastfeeding**	**β crude**	**95% CI**	*p-value*[2]	**β adjusted**[3]	**95% CI**	*p-value*[2]
Weight for age at 5 months <2.5 SD	≤ 2 months	0.17	(0.11,0.23)		0.03	(-0.02, 0.09)	
	>2- <4 months	0.01	(-0.03, 0.06)		-0.01	(-0.05, 0.03)	
	≥ 4 months	0.00	-		0.00	-	
Weight for age at 5 months ≥2.5 SD	≤ 2 months	0.74	(0.45, 1.04)		0.50	(0.22, 0.77)	
	>2- <4 months	0.71	(0.53, 0.89)		0.51	(0.34, 0.68)	
	≥ 4 months	0.57	(0.32, 0.82)	0.67	0.40	(0.16, 0.63)	0.68

[1]BMI z-score was calculated according to the LMS method, [2]P-values are for the between duration of exclusive breastfeeding and infant weight, [3] Adjusted for: Maternal pre-pregnancy BMI, paternal BMI, maternal smoking during pregnancy (grams of tobacco per day), maternal physical activity during pregnancy (3 levels), weekly gestational weight gain, socio-economic status of the mother (3 levels), child sex and birth weight (continuous).
