# Supplementary material for: Overweight in childhood of exclusively breastfed infants with a high weight at 5 months
Source: Matern Child Nutr. 2020 Aug 20;17(1):e13057. doi: 10.1111/mcn.13057 (PMC7729543; doi:10.1111/mcn.13057)
Supplement: Supplementary file 5 — Table S5. Associations between duration of exclusive breastfeeding, overweight1 and BMI z‐score at age 7 years for infants ≥2.5 SD weight for age at 5 months, n = 316. [file MCN-17-e13057-s005.pdf]

Supplementary Table 5. Associations between duration of exclusive breastfeeding, overweight[1] and BMI z-score at age 7 years for infants ≥2.5 SD weight for age at 5 months, n=316.

Exposure breastfeeding	OR crude	95% CI	OR adjusted[2,3]	95% CI
≤2 months	1.31	(0.66, 2.59)	1.05	(0.48, 2.27)
>2- <4 months	1.06	(0.59, 1.90)	1.01	(0.54, 1.91)
≥4 months	1.00	-	1.00	-
	**β crude**	**95% CI**	**β adjusted[2,3]**	**95% CI**
≤2 months	0.29	(-0.01, 0.60)	0.14	(-0.16, 0.43)
>2- <4 months	0.04	(-0.21, 0.30)	0.00	(-0.23, 0.24)
≥4 months	0.00	-	0.00	-

[1]Overweight is categorized according to the International Obesity Task Force reference, [2]Adjusted for: maternal pre-pregnancy BMI, paternal BMI, maternal smoking during pregnancy (continuous), maternal physical activity during pregnancy (3 levels), weekly gestational weight gain, socio-economic status of the mother (3 levels) child sex and birth weight (continuous), [3]Test for trend was non-significant, p>0.05.
