# Supplementary material for: Overweight in childhood of exclusively breastfed infants with a high weight at 5 months
Source: Matern Child Nutr. 2020 Aug 20;17(1):e13057. doi: 10.1111/mcn.13057 (PMC7729543; doi:10.1111/mcn.13057)
Supplement: Supplementary file 6 — Table S6. Associations between duration of exclusive breastfeeding, overweight1 and BMI z‐score at age 11 years for infants ≥2.5 SD weight for age at 5 months, n = 214. [file MCN-17-e13057-s006.pdf]

Supplementary Table 6. Associations between duration of exclusive breastfeeding, overweight[1] and BMI z-score at age 11 years for infants ≥2.5 SD weight for age at 5 months, n=214.

Exposure Breastfeeding	OR crude	95% CI	OR adjusted[2,3]	95% CI
≤2 months	1.05	(0.48, 2.27)	0.60	(0.17, 2.08)
>2- <4 months	1.02	(0.54, 1.91)	0.84	(0.33, 2.14)
≥4 months	1.00	-	1.00	-
	**β crude**	**95% CI**	**β adjusted[2,3]**	**95% CI**
≤2 months	0.17	(-0.17, 0.51)	0.10	(-0.20, 0.40)
>2- <4 months	0.13	(-0.14, 0.40)	0.12	(-0.12, 0.35)
≥4 months	0.00	-	0.00	-

[1]Overweight is categorized according to the International Obesity Taskforce reference, [2]Adjusted for: maternal pre-pregnancy BMI, paternal BMI, maternal smoking during pregnancy (continuous), maternal physical activity during pregnancy (3 levels), weekly gestational weight gain, socio-economic status of the mother (3 levels) child sex and birth weight, [3]Test for trend was non-significant, p>0.05.
